# Scientific clickbait: Examining media coverage and readability in genome-wide association research

**DOI:** 10.1371/journal.pone.0296323

**Published:** 2024-01-05

**Authors:** José J. Morosoli, Lucía Colodro-Conde, Fiona Kate Barlow, Sarah E. Medland

**Affiliations:** 1 Mental Health & Neuroscience Department, QIMR Berghofer Medical Research Institute, Brisbane, QLD, Australia; 2 School of Psychology, University of Queensland, Brisbane, QLD, Australia; Max Planck Institute for Solid State Research, GERMANY

## Abstract

In the present study, we analyzed a large corpus of English-language online media articles covering genome-wide association studies (GWAS), exemplifying the use of computational methods to study science communication in biological sciences. We analyzed trends in media coverage, readability, themes, and mentions of ethical and social issues, in over 5,000 websites published from 2005 to 2018 from 3,555 GWAS publications on 1,943 different traits, identified via GWAS Catalog using a text-mining approach to inform the discussion about genetic literacy and media coverage. We found that 22.9% of GWAS papers received media attention but most were described in language too complex to be understood by the public. Ethical issues are rarely mentioned and mentions of translation are increasing over time. We predicted media attention based on year of publication, number of genetic associations identified, study sample size, and journal impact factor, using a regression model (r^2^ = 38.7%). We found that chronotype, educational attainment, alcohol and coffee consumption, sexual orientation, tanning, and hair color received substantially more attention than predicted by the regression model. We also evaluated the prevalence of the clickbait “one gene, one disease” headlines (e.g., “Scientists Say They’ve Found Gene That Causes Breast Cancer”) and found that it is declining. In sum, online media coverage of GWAS should be more accessible, introduce more modern genetics terms, and when appropriate, ELSI should be mentioned. Science communication research can benefit from big data and text-mining techniques which allow us to study trends and changes in coverage trends across thousands of media outlets. Results can be explored interactively in a website we have built for this manuscript: https://jjmorosoli.shinyapps.io/newas/.

## Introduction

Over the last few decades, online news and media have become the main source of scientific information for many individuals and decision-makers [1, 2]. From university press releases and in-depth journalistic articles on new scientific publications to BuzzFeed-style short pieces, people have added the internet to their toolbox to better understand the world around them, pushing both science communicators and scientists to learn how to convey science outputs across a variety of audiences effectively [3], which becomes especially relevant when it comes to communicating findings with meaningful social implications that can potentially divide public opinion, such as global warming, artificial intelligence or human genetics [4]. For these and other reasons, the study of media coverage of science has become one of the central topics for social scientists [5] and in recent years more and more studies evaluating the nuances of scientific communication have been published [4, 6, 7].

Among the prerequisites for effective scientific communication is that the content is readable and understandable by the intended audience. Overuse of technical language or *jargon*, and low *readability* are potential barriers to any kind of communication attempt. On the one hand, *readability* refers to how easy to understand a text is, and it depends on the content, style, design, and organization with prior knowledge, reading skill, interest, and motivation of the intended audience [8], and it has traditionally been measured using *readability formulas*. These formulas use vocabulary range and sentence length to predict text difficulty and estimate the level of reading skill required to understand it [8]. On the other hand, *jargon* comprises special words or expressions used by a profession or group that are difficult for others to understand, and undermine efforts to inform and persuade the public [9, 10]. In addition, the interpretation of information can also be influenced by the presence or absence of certain words, phrases, or images in an article through a mechanism known as *framing* [2]. Effective science communication also involves the use of framing in a way that overcomes audience heuristics and personal motives that interfere with an accurate understanding of scientific knowledge [11, 12], or in the case of genetics, prevent deterministic understanding of genetic influence [13].

In the past, studies on media coverage of science have focused on printed media. Across scientific disciplines, only 2.7% of 337 studies on media coverage of science between 1956 and 2009 analyzed Internet media [5]. To the best of our knowledge, no systematic review has been conducted on this topic since 2010, but we expect this percentage to have increased over the last decade, especially due to the increased availability of big data and computational methods to analyze online media [14]. For example, recent publications have explored the relationship between science communication and Reddit [15], Twitter [16], and YouTube [17], as well as the relationship between press releases and news articles [18]. However, despite a growing body of studies analyzing online media, the enormous amount of online content generated daily still poses a challenge to most researchers who are interested in analyzing media coverage of science [19]. Big data and computational methods facilitate this task by allowing researchers to analyze large amounts of data in a time-efficient way, providing in some cases more generalizable evidence relative to qualitative and small-scale quantitative studies while identifying new trends and patterns and insights about the bigger picture [5, 20]. Some of the few applications of computational techniques for digital news outlets, such as text mining, include COVID-19 vaccine coverage [21], understanding public health concerns [22] and identifying the scientific topic areas most prevalent in mass media in general [23]. While the use of computational methods in environmental communication has recently gained attention [14, 19], their use for the study of online mass media coverage of biological sciences in general, and human genetics in particular, is rare [23].

Previous research on media coverage of human genetics has focused almost exclusively on printed media and showed that in the past, the portrayal of genetics in the news has been simplistic, it often disregarded failures to replicate findings, promoted the nature *vs* nurture dichotomy, and ignored ethical challenges [24–27] despite concerns about the implications of genetic findings (i.e., privacy, insurance coverage, and discrimination) being widespread [28], while finding heterogeneous levels of genetic determinism [27, 29, 30]. Other barriers to effective science communication, such as readability or the use of jargon, have received little attention. Moreover, after overcoming multiple of their initial limitations, genome-wide association studies (GWAS) have been able to provide meaningful insights into disease biology and behavior and teach us about the effects of natural selection and adaptation, expose causal relationships between risk factors and disease, help identify new drugs for common disorders, produce polygenic scores currently undergoing clinical trials, and even complement social science research [31]. GWAS and their applications are very likely to remain in the scientific landscape for the foreseeable future and will likely continue to pose communication challenges that need to be addressed. Therefore, in the present study, we analyzed a large corpus of English-language online media articles covering GWAS, exemplifying the use of computational methods to study science communication in biological sciences [14, 19]. In particular, we aimed to achieve the following objectives:

1. To analyze **readability and use of jargon** in media coverage of GWAS using text mining.
2. To evaluate the **framing of genetic findings** in the news by combining text mining with an already-existent gene-related framing scheme.
3. To quantify **mentions of ethical, legal, and social issues (ELSI) keywords**.
4. To identify the most common topics in media coverage of GWAS using unsupervised **topic modeling**.

Additionally, we also tested the claim that science journalism largely relies on measures of relevance provided by science itself to choose *which publications to cover* [1] by **predicting media attention** to GWAS based on the number of significant genetic associations reported, year of publication, sample size, journal impact factor, etc.

## Method

### Identifying GWAS publications

We retrieved PubMed identifiers (PMIDs) and citation metadata from all GWAS publications indexed by the NHGRI-EBI GWAS Catalog by 17 September 2018 [32]. GWAS Catalog identifies eligible studies by literature search and assessed by in-house curators, who then extract the reported trait, significant gene-trait associations, and sample metadata (e.g., sample size). Studies are included in the GWAS Catalog database within 1–2 months of publication, dependent on the availability of literature, and the data is released on a weekly cycle. Eligible studies must include a primary GWA analysis, defined as array-based genotyping covering variation across the whole genome, the study was published in English, and the study includes new data. GWAS Catalog was originally founded by the National Human Genome Research Institute (USA) and is currently maintained by European Bioinformatics Institute. The reported trait of each GWAS was manually classified into non-disease and disease traits using the International Classification of Diseases 10^th^ Revision (ICD-10) [33]. Disease traits were further classified into each associated chapter of the ICD-10 (e.g., neoplasms, mental and behavioral disorders, etc.). Our dataset included 3,555 unique PMIDs to GWAS publications on 1,943 different traits.

### Retrieving online mentions of scholarly articles

Online mentions of publications were identified via a research agreement with Altmetric [34]. Altmetric tracks online attention to research publications by automatically recognizing the use of unique identifiers (e.g., PubMed ID, DOIs, ISBNs) in a wide range of online documents, including public policy documents, mainstream media, social media, blogs, patents and more. Altmetric tracks mentions of papers in the news by (i) searching for a direct hyperlink to a scholarly paper in the content of a news report, and (ii) searching the news report’s text for mentions of the scholarly paper, journal, and author(s). We queried the Altmetric Explorer API [34] by searching for mentions of the 3,555 unique PubMed IDs obtained from the GWAS Catalog to identify news sites and blogs that had mentioned any of those GWAS publications. From Altmetric Explorer API we obtained a list of URLs to every news site and blog that mentioned any PMID in our query, as well as metadata from the news site and blogs (i.e., date of publication, language, name of media outlet). We decided to use Altmetric as our database for media attention because it has been shown to have better coverage of blogs, news, and tweets, surpassing the other providers, among other advantages [35, 36].

**Fig 1:**
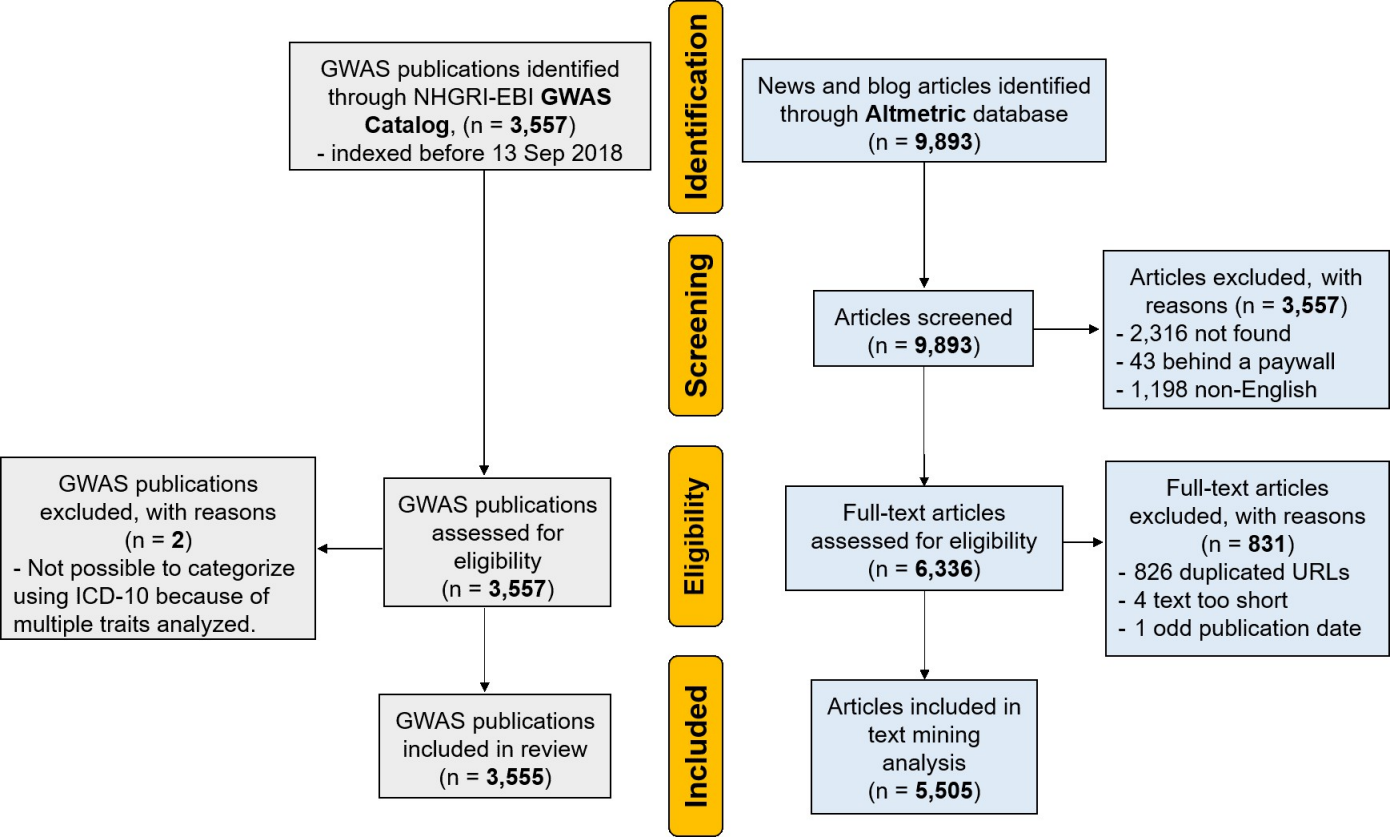
Online review flow diagram. Duplicated URLs refer to news sites and blogs that are linked to more than one GWAS publication. In those cases, we only analyzed the website once. In the case of identical or almost identical websites (identical, aggregated, or rephrased), we analyzed all entries.

Each URL was accessed and coded as ‘found’ or ‘not found’ and reasons were reported (see S4 Table in S1 File). After excluding duplicated news sites and blogs, our text corpus consisted of 5,505 English-language online news sites (see Fig 1 for more details and eligibility criteria). Text in the online mentions was retrieved by hand, stored as a text file, and analyzed using *tidytext* [37]. Data analysis was conducted in R-3.6.2 [38]. We built an interactive web app using *shiny* in R [39] where more in-depth results and interactive visualization can be found [40]. Potential bias selection bias was addressed by screening all GWAS publications available to date, as well as all news sites collected by Altmetric by 17 September 2018. Details about the GWAS publications and online references reviewed can be found in S1-S5 Tables in S1 File. Original text files and descriptive statistics can be found in S1 Dataset. More details about which sources are tracked by Altmetric can be found at https://www.altmetric.com/about-our-data/our-sources/news/.

### Readability and use of jargon

To provide a baseline of language complexity of online media, we calculated four readability indexes for each news site and blog: the Flesch Reading Ease score (FRES), Flesch-Kincaid Grade Level (F-K), Gunning Fog Index (GFO), and Simple Measure of Gobbledygook (SMOG). For a description of formulas, see Main results - Readability at https://jjmorosoli.shinyapps.io/newas/. In short, GFI estimates the years of formal education a person needs to understand the text on the first reading; SMOG estimates the years of education needed to understand a piece of writing; higher FRES scores indicate material that is easier to read and passages that are more difficult to read have lower scores; and F-K presents readability scores as the US grade level required to understand the text and it can also be interpreted as the number of years of education generally required to understand a text. Finally, to estimate the prevalence of genetic jargon in media coverage of GWAS, we calculated how many of the terms from the NHGRI Talking Genetics Glossary [41] were present in each article.

### Framing of genetic findings

To identify the frames used in the news articles, we analyzed the use of key terms using an adaption of the framework developed by Carver et al. [13] which classifies representations of genetics across traits and time as *materialistic*, *deterministic*, *relativistic*, *evolutionary*, or *symbolic* (see Box 1). In their framing scheme, Carver et al. provided a list of keywords and phrases associated with each frame. For example, a deterministic frame is associated with titles such as ‘The gene for depression’ and keywords such as ‘cause’, ‘control’, ‘blame’ and ‘disease’. The framing scheme developed by Carver and colleagues provides a transparent and straightforward way of classifying gene discourse. It can be applied to the analysis of any type of gene-related communication (i.e., textbooks, classroom materials, governmental reports, or news articles) and it forms a basis for quantitative analysis, allowing researchers “to identify the ‘gene profile’ of a particular newspaper”.

**Box 1. Gene framing scheme, modified from Carver, Wiese [42]. Asterisks indicate that all derived words from that root word were included. Note: symbolic and deterministic frames were corrected for negation words (e.g., ‘not in your genes’ was not scored towards a deterministic frame)**

**Table:** 

**Gene frame**	**Description**	**Keywords and phrases**
1. Materialistic	A discrete physical unit	Identif*, Locate, Isolate, Transfer, Specific, Replace, Inject, Discover, Code.
2. Deterministic	A definite causal agent	Gene for, Cause, Control, Culprit, Disease-gene, Responsible for, Wired in, Born with, Genes or environment, Down to our gene*, Born this way.
3. Relativistic	A predisposing factor	Risk, Chance, Factor, Associated with, Susceptible to, Linked to, Contribut*, Predispos*, Interfer*, Influence, Play a part in, Plays a part in, Genes are involved.
4. Evolutionary	A dynamic agent interacting with the environment	Natural selection, Make copies, Replicate, Reproduc*, Through generations, Adapt, Maladapt, Evol*, Relatedness, Conserv*, Diversity, Development, DNA record, Gene bank, Marker, Extinct, Change, Interact*, Complex, Dynamic, Capacity, External influence, Environment, Depends on, In combination with, Affected by, Expression, Triggered by, Prevent, Respond, Turn on, Turn off.
5. Symbolic	An abstract representation of relationship	In the / my / your / their genes.

Carver et al., however, did not develop their framing scheme necessarily with text mining in mind. Instead of manually coding the presence of gene frames in each news and blog article–the methodology used by Carver et al in the first application of their gene-frame scheme [30], we develop a novel algorithm to calculate the relative usage of each frame in any particular text. We estimated the relative usage of frames in an article by dividing the proportion of key terms of a specific frame by the weighted sum of total key terms of all five frames (Box 1) present in that article within an article using an equation developed by us, which calculates relative usage of frames in a text mining-friendly fashion (see Formula 1).

$$\frac{\frac{n_{i,j}}{m_{j}}}{\sum_{j=1}^{5}\frac{n_{i}}{m_{j}}}∙100$$ (1)

Where *n* is the number of keywords of *j* frame (i.e., materialistic, deterministic, relativistic, evolutionary, or symbolic) present in a given news article *i*, and *m* is the maximum possible number of keywords for frame *j* out of 5. Please note that symbolic and deterministic frames were corrected for negations (e.g., ‘not in your genes’ was not scored towards a deterministic frame). Therefore, our approach assumes that the more key terms associated with a specific frame that are used in a given article, the more likely that frame represents how the journalist decided to describe the gene. For an example see Box 2.

**Box 2. Example. News article no. 614**

**Table:** 

**Calculate proportion of keywords present in the article for each frame:** *n*_614,materialistic_ *=* 1 (out of 13) *n*_614,mat_/*m*_mat_ = 1/13 = 0.077 *n*_614,deterministic_ = 0 (out of 11) *n*_614,det_/*m*_det_ = 0/11 = 0 *n*_614,relativistic_ = 2 (out of 12) *n*_614,rel_/*m*_rel_ = 2/12 = 0.167 *n*_614,evolutionary_ = 1 (out of 32) *n*_614,evo_/*m*_evo_ = 1/32 = 0.031 *n*_614,symbolic_ = 0 (out of 5) *n*_614,sym_/*m*_sym_ = 0/5 = 0 **Calculate total usage:** *∑n*_614, j_*/m*_j_ *=* 0.077 + 0 + 0.167 + 0.031 + 0 = 0.275	**Calculate relative usage:** Materialistic = 0.077 / 0.275 ∙ 100 = 28.0% Deterministic = 0 / 0.275 ∙ 100 = 0% Relativistic = 0.0167 / 0.275 ∙ 100 = 60.6% Evolutionary = 0.031 / 0.275 ∙ 100 = 11.4% Symbolic = 0 / 0.275 ∙ 100 = 11.4%

We conducted two additional analyses to further evaluate the framing of genetic findings in media coverage of scholarly articles: (i) we evaluated the frequency of positive and negative terms in the news corpus using the *tidytext* package [37] and combining terns from the Bing, NRC and AFINN sentiment lexicons [43]; and (ii) we computed how many times the expression “gene for” was used in headlines and bodies of news, as a proxy for the presence of the “one gene, one disease” frame in the media, that is, the idea that a single gene determines the disease or trait (e.g., “Scientists Say They’ve Found Gene That Causes Breast Cancer”) [26].

The frequency of positive and negative terms was accounted for negation (i.e., reversed sentiment when the term was preceded by "not", "without", "no", "can’t", "don’t", or "won’t"). The validity of searching for the term ‘gene for’ to capture the use of true “one gene, one disease” statements in the media was evaluated by manually inspecting each headline and paragraph where the term “gene for” was found. Each match was then classified into (a) true oversimplifications or (b) correct uses of the term ‘gene for’.

### Mention of ELSI keywords

We analyzed the frequency of use of terms associated with the translation of genetic research and ELSI within news coverage, across time and traits by searching for 13 terms (e.g., ‘ethic’, ‘policy’, ‘discrimination’; see S5 Table in S1 File for the full list of terms). The list of terms was curated by the research team upon evaluating common terms in the literature around this topic.

### Topic modeling

We used unsupervised structural topic modeling to identify overarching themes in news coverage. Topic modeling classifies words into natural categories based on their co-occurrence within a document. In the online news analyzed, a model with 30 topics showed the best fit. We used the *stm* package [44] to implement structural topic modeling and identify latent topics in our text corpus. The optimum number of latent topics was decided based on (i) the highest held-out likelihood and semantic coherence and (ii) the lowest residuals, which led us to choose a 30 topics solution [45]. Individual listings of publications and online mentions are reported in S1-S4 Tables in S1 File. Results are also accessible on our interactive website: https://jjmorosoli.shinyapps.io/newas/.

### Predicting media attention

We combined the metadata from GWAS Catalog and the Journal Citation Reports [46] and we used negative binomial regression analyses to evaluate if the number of online mentions of GWAS publications could be predicted by (i) year of publication, (ii) number of significant loci, (iii) discovery sample size, and (iv) journal impact factor. The dependent variable ‘online mentions’ was based on the number of online mentions originally identified by Altmetric (including news articles in languages other than English for which we were not able to retrieve the text; see Fig 1). Journal impact factor for GWAS publications was defined as the impact factor in the year before the paper was published. The distribution of the dependent variable ‘online mentions’ was highly skewed, and a negative binomial regression model was preferred over a linear model. Regression analysis was conducted using *glmmTMB* [47]. When publications came from journals with no impact factor, the impact factor was assumed to be zero, otherwise, there was no missing data. Secondary data used in these analyses can be found in S2 Dataset.

## Results

### Description of the dataset

**Fig 2:**
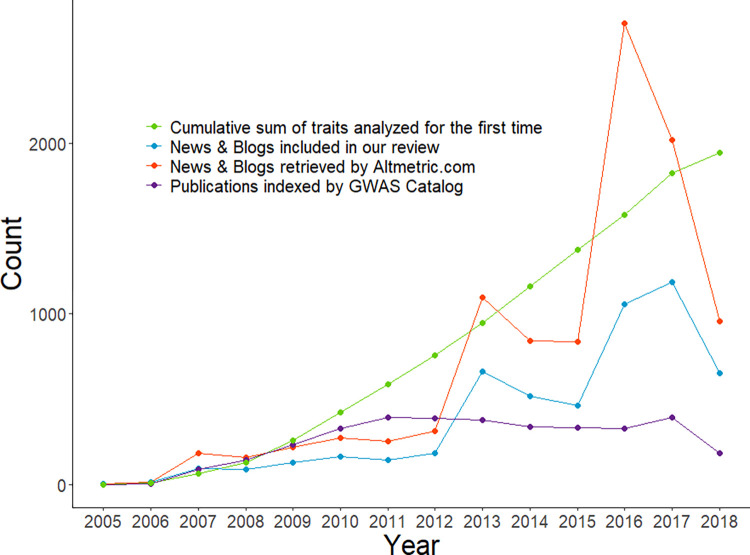
Number of GWAS publications and mentions in news and blogs by year. Online attention to GWAS has increased over time independently from the increase of GWAS publications per year (partial r = 0.69 [0.23,0.90]).

Only 22.9% of published GWAS were reported online. Almost 40% of retrieved news and blog articles contained identical, aggregated, or rephrased content (i.e., correlation between word frequencies higher than *r* = 0.95) from another website. Both GWAS publications and their online coverage increased each year (see Fig 2). The most frequently studied traits have been non-disease traits (33.7%), neoplasms (13.0%), and mental and behavioral disorders (10.4%). These were also the traits most frequently covered in the news, receiving 43.4%, 11.6%, and 14.1% of all news reports, respectively.

### Readability and use of jargon

Across readability indexes, analyses showed that 95% of the news sites and blogs would require the reading ability of a university student (i.e., more than 12 years of formal education). General online media such as that produced by The Huffington Post or CNN is approximately five times less complex [48; see Table 1].

**Table 1:** Comparison of average readability scores across different sources.

Source	GFI	SMOG	FRES	F-K
News & blogs on GWAS	21	15.5	-108.2	31.3
BuzzFeed Top 500 (2016) [48]	3.1	3.8	58.7	5.6
HuffPost Top 500 (2016) [48]	5	5	N/A	5
CNN Top 500 (2016) [48]	7	6	N/A	6
Health websites (Australia) [49]	N/A	12.1	47.5	10.5

*Note*: GFI = Gunning fog index; SMOG = Simple Measure of Gobbledygook; FRES = Flesch reading-ease score; F-K = Flesch–Kincaid grade level.

In terms of use of jargon, the 5 most common genetic terms across all websites were RNA (which was present in 65.3% of websites), risk (63.7%), gene (62.0%), genome (61.1%), and DNA (45.0%). On average, each website used 9 out of 231 terms present in the NHGRI Talking Genetics Glossary (M = 8.8, SD = 4.5). Note that ‘risk’ was used in 63.7% of news articles versus ‘susceptibility’ (12.2%) or ‘protect’ (11.3%); and ‘gene’ was used in 62.0% while ‘marker, ‘polymorphism’, or ‘allele’, where used in 15.9%, 11.6%, and 6.3% respectively. Core terms in complex trait genetics, such as ‘polygenic’ and ‘interaction’, only appeared in 2.9% and 6.7% of all news articles, respectively.

### Framing of genetics findings

**Fig 3:**
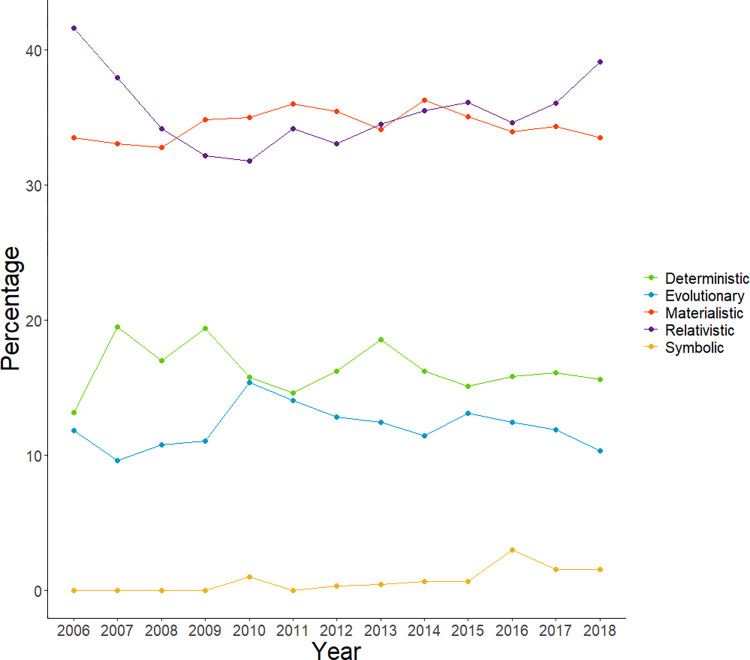
Relative use of frames over time across all traits. Data points indicate the average presence of frames within that year across all news sites and blogs.

Relativistic and materialistic frames were the most frequent frames used in news sites and blogs. The use of deterministic keywords and phrases was comparatively lower. This pattern is stable across time, although in mental and behavioral disorders we observe a decrease in the use of deterministic terms and an increase in relativistic terms. In summary, we found that articles used predominantly a relativistic frame (35.8%), followed by a materialistic frame (34.4%) and to a lesser extent a deterministic (16.2%) and evolutionary frame (12%). See Fig 3 and our website [40] for more details.

When it comes to the use of the “one gene, one disease” model in the media, we found that it was used in 23 headlines, and 160 times in 74 of the news bodies. After inspecting the latter: 50 were true OGOD simplifications, 66 were claims against the idea of OGOD, and 46 were correct uses of the term ‘gene for’. True OGOD statements were more common in coverage of genetic studies on gray hair (N = 26), intelligence (N = 4), and twinning (N = 3); while claims against it were more common in coverage of educational attainment (N = 15), personality (N = 6), and depression (N = 6). Moreover, the phrase ‘*gene for*’ was rarely used between 2005 and 2012, but appeared more frequently from 2013 onwards [40]. Finally, positive words were used more frequently than negative words, although the prevalence of both positive and negative terms was low, suggesting that news coverage was not typically emotionally valenced. The most common positive adjectives were ‘novel’ (used in 14.7% of news articles), ‘unique’ (6.8%), and ‘robust’ (4.4%), while the most common negative adjectives were ‘weak’ (7.6%), ‘ineffective’ (0.8%), and ‘inadequate’ (0.7%).

### Mention of ELSI keywords

We found that mentions of clinical implications in GWAS coverage have increased over time. The terms ‘prevent’ (24.8% of 5,505 news articles), ‘therap*’ (23.7%), ‘screening’ (7.4%), ‘precision medicine’ (2.4%), ‘detection’ (2.3%), and ‘pharmacogenomics’ (0.9%), all started to appear more frequently from 2015 onwards (40). However, there was relatively little change in the use of the terms related to ELSI, such as ‘policy’ (2.7% of news articles), ‘*ethic*’ (2.1%), ‘minorit*’ (1.9%), ‘privacy’ (1.4%), ‘stigma’ (1.2%), ‘discrimination’ (0.9%), ‘insurance’ (0.7%), and ‘eugenic*’ (0.9%), which remained low over the years [40].

### Topic modeling

Finally, in our news corpus, a model with 30 topics showed the best fit. The top five topics in the news coverage were major depression, cancer in women, asthma and empathy, and educational attainment (see Table 2). Note that topic modeling algorithms do not provide a label for each topic. We give meaning to each topic by arbitrarily labeling them based on most common words within each topic. In the case of “asthma and empathy”, no other label seemed appropriate to subsume those keywords. Next, we classified each article based on the topic it belonged to, which allowed us to explore the context in which the keywords and frames described above were used. The topic ‘sleep disorders’ had the highest use of deterministic keywords, and ‘major depression’ had the lowest. The word ‘environment’ was most frequently used when talking about ‘immune system’ and ‘educational attainment’, while ‘eugenic’ was most frequently used with the topics ‘educational attainment’ and ‘cancer in women’.

**Table 2:** Top 10 topics in the GWAS news corpus.

Topic	Top 7 terms that contribute to each topic
*Major Depression*	Depression, disorder, scientists, university, condition, psychiatric, major
*Cancer in women*	Cancer, breast, lung, women, ovarian, cancers, common
*Methods*	SNP, association, loci, wide, analysis, studies
*Asthma and empathy*	Women, empathy, twins, birth, children, asthma, age
*Educational attainment*	Education, intelligence, differences, social, attainment, twins, environment
*Facial genetics*	Hair, skin, nose, facial, color, shape, pigmentation
*Ancestry*	African, American, ancestry, European, populations, variant, children
*Alzheimer’s disease*	Brain, Alzheimer’s, cognitive, memory, dementia, found, scientists
*Diabetes*	Diabetes, obesity, type, life, lifespan, health, diseases
*Cardiovascular disease*	Heart, blood, stroke, pressure, cholesterol, cardiovascular, coronary

*Note*: Labels were manually assigned based on top terms within each topic.

### Predicting media attention

More recent publications (Incidence rate ratio (IRR) = 1.88 [1.71, 2.06], P < .001), with bigger sample sizes (IRR = 2.34 [1.83, 3.05], P < .001), and published in journals with higher impact factors (IRR = 2.67 [2.39, 3.01], P < .001), received more media attention. In regards to significant interactions with publication year we found that in later years, sample size was less predictive of coverage (IRR = 0.69 [0.57, 0.83], P < 0.001) while impact factor was more predictive (IRR = 1.12 [1.02, 1.23], P = 0.015). There was also a significant interaction between impact factor and sample size (IRR = 0.87 [0.77, 0.97], P = 0.003). The regression model explains 38.7% of the variance. Examination of residuals showed that while the model was accurate for most traits, neoplasms, behavioral disorders, chronotypes, intelligence and educational attainment, and alcohol and coffee consumption, received substantially more online mentions than predicted by our model, suggesting differential trends in media interest. Comprehensive results from the regression analyses are available on our website [40].

## Discussion

In the present study, we analyzed a large corpus of English-language online media articles covering genome-wide association studies, exemplifying the use of computational methods to study science communication in biological sciences. Our results show that new genetic research is described in language too complex to be understood by the public. That is, guidelines for effective communication suggest aiming for two to five grades lower than the highest average grade level of your intended audience [50]. However, over 95% of the news sites and blogs would require more than 12 years of formal education. For example, if we place this in a US context, about 46.3% of US adults hold an associate’s degree or higher and in 2018, only 6.3% of those with a bachelor’s degree in the US majored in biological, agricultural, or environmental sciences–degrees that might feasibly introduce people to genetic terminology [51], meaning that online coverage of GWAS might be effectively inaccessible for approximately 64% of US adults [2]. We encourage journalists and science communicators to run basic readability analyses for the media articles before publishing them and aim for a complexity level similar to those of general news outlets (e.g., the reading ability of a person with a high school certificate).

Next, we identified the most common technical terms used in media coverage of genetics and found that on average, each website used 9 out of 231 terms included in the NHGRI Talking Genetics Glossary, suggesting a low use of jargon. This may reflect an effort to make genetic research more accessible; however, the challenge lies in finding the right balance between using more readable language and introducing contemporary genetic terminology. That is, eliminating jargon may not lead to more accessible science communication accessible and can remove the opportunity to explain more complex genetic concepts to readers. The fact that core terms in complex trait genetics, such as ‘polygenic’ and ‘interaction’, only appeared in 2.9% and 6.7% of all news articles, respectively, might indicate that at least when it comes to covering new GWAS findings, we are failing to introduce more nuanced but essential concepts in modern genetics.

In regards to how genetic findings are framed, our results are somewhat similar to a previous review analyzing news content about genetics published in tabloid and elite newspapers in 2005–2008 by the original authors of the gene-framing scheme [30]. We found similar trends in the usage of deterministic (16.2%) and evolutionary frames (12% *vs* 12.9%) but higher average usage of relativistic (35.8% *vs* 13.5%) and materialistic frames (34.4% *vs* 25.6%). While these results might indicate a switch in media towards a more nuanced and descriptive coverage of genetic research, previous work has found that genetic explanations of human behavior often activate deterministic assumptions [52, 53]. That is, despite the relatively low presence of deterministic frames, a single deterministic catchphrase might override complex explanations of genetics, especially given the low readability of news coverage on GWAS. Additionally, the application of a framing scheme in a text-mining context will require a specific empirical study evaluating the reliability of text-mining algorithms in general, and of our relative usage of frames approach.

The next finding from our study is that ethical issues are largely unaddressed, while suggestions for translation are increasing over time. Given public concerns about privacy and potential discrimination [28], researchers might consider mentioning ELSI more often when reporting genetic findings given that it is desirable to engage early with the public when working on topics that have the potential to become contentious [2]. Previous qualitative research has shown that when it comes to more applied genetic research, such as predictive genetic testing, big media outlets such as The Guardian and the Daily Telegraph do cover most of the ethical issues identified in the scientific and ethical literature, but also find a similar proportion to the ones observed by us of articles mentioning discrimination and stigma [54]. While the need to mention ELSI in media coverage of GWAS is debatable, the aforementioned lack of use of more nuanced genetic terms such as polygenic and interaction means that we are most likely missing the opportunity to use media reporting to introduce the necessary terms for a realistic understanding of modern genetics [55].

Regarding the most common topics in media coverage of GWAS findings, our results show that both disease (cancer, mental health, cardiovascular disease) and non-disease traits (facial genetics, educational attainment, ancestry) are major themes in media coverage and when it comes to predicting media attention, the fact that our regression model explains 38.7% of the variance supports the notion that a measure of relevance, such as impact factor, influences which stories get covered, and that this has become more salient in recent years. While this finding is not surprising, the present study allows us to have a baseline of previous and current trends in media coverage of genetic research. It also shows that impact factor has become more and more important when it comes to which studies receive media attention, which echoes findings such as impact factor predicting the number of citations above and beyond study methodology and design [56], and newspaper coverage predicting future citation count of scientific publications [57]. The growing use of impact factor metrics beyond their intended purpose [58] must be further explored. The scientific community should make sure that there are guides and educational material available to journalists and science communicators to evaluate the quality of a study, independently from overly simplistic measures of quality, such as journal impact factor.

### Limitations

There are several limitations to our work. First, we have focused on GWAS and have not compared this to other scientific areas. GWAS is a relatively recent and widely used technique which makes it difficult to find a technique of a similar age and popularity to use as a comparison. Similar studies targeting other techniques or scientific fields would strengthen the value and interpretability of the approach used in the present study. Second, a text mining method does not allow us to determine if technical terms are explained or not–this question still requires a manual review of selected articles. Third, the scope of this review was limited to English language. Applying text mining to non-English languages poses challenges due to linguistic diversity, limited resources, and potential inaccuracies in machine translation. Cultural nuances and language-specific complexities, such as morphological variations, can impact sentiment analysis and topic modeling as well. Additionally, syntax differences, data sparsity, and lack of standardized evaluation hinder effective adaptation and performance assessment of text mining techniques for non-English texts. While the methodology presented in the present study can be easily applied to other text corpora, future studies should aim to replicate these findings using non-English language websites. Fourth, our results are limited to news articles that cite original research works. This allows us to be very precise about media attention to a particular field of study, but in turn, it means that our review does not provide information on how people talk about genetic research when no reference to a scholarly publication is made. Nevertheless, we argue that our approach does inform science communication campaigns that come from professional science journalists and research institutions, who are most likely to be citing research papers in their news pieces. Fifth, our review is limited to media sources monitored by Altmetric. However, even if Altmetric coverage is not exhaustive, it is extensive and covers major national and international media sources, including the most well-known written newspapers. Sixth, analyzing the frequency of specific words as a proxy for framing analysis, despite providing more objectivity to the process, oversimplifies the qualitative analysis of content framing. While we argue that combining a strong theory with text-mining has the potential to adapt framing analysis to big data, the algorithm presented in this study requires further validation where the presence of gene-frames is independently evaluated by human coders and text-mining algorithm in the same subset of texts. Finally, our sentiment analysis does not capture the context in which words are used, only if they were preceded by a negation. Although the prevalence of emotional language was low, future studies could investigate distinguishing negative and positive mentions to evaluate the valence of the news articles.

## Conclusion

In conclusion, in the first text-mining review of online media coverage of genetic studies, we characterized the use of technical vocabulary, themes, emotional valence, and topics in human genetics, among others. We also identified potential barriers to effective communication: online media coverage of GWAS should be written so it is more accessible, introduce more modern genetics terms, and when appropriate ELSI should be mentioned. We argue that science communication research in our field can benefit from big data and text-mining techniques which can be used to regularly monitor trends and changes in coverage trends across thousands of media outlets, allowing us to monitor and improve communication practices in a fast-evolving online media landscape.

## Supporting information

S1 Dataset Text body and metrics of news and blog articles included in the manuscript. (ZIP)

S2 Dataset PMID and metadata of research articles included in the manuscript. (ZIP)

S1 File Inclusions and exclusion (research articles, news and blogs and terms) with rationale. (ZIP)

## References

[1] Schäfer MS. How changing media structures are affecting science news coverage. The Oxford Handbook of the Science of Science Communication 2017. p. 51–7.

[2] National Academies of Sciences Engineering and Medicine. Communicating Science Effectively: A Research Agenda. Washington, DC: The National Academies Press; 2017. 28406600

[3] Holub A. Communicating science in an age of on-screen reading: taking a page from journalism. Journal of Epidemiology and Community Health. 2020;74(9):754–6. doi: 10.1136/jech-2019-213257 32507750

[4] Morosoli JJ, Colodro-Conde L, Barlow FK, Medland SE. Public Understanding of Behavioral Genetics: Integrating Heuristic Thinking, Motivated Reasoning and Planned Social Change Theories for Better Communication Strategies. Behavior genetics. 2019;49(5):469–77. doi: 10.1007/s10519-019-09964-9 31317344

[5] Schäfer MS. Taking stock: A meta-analysis of studies on the media’s coverage of science. Public Understanding of Science. 2010;21(6):650–63. doi: 10.1177/0963662510387559 23832152

[6] Donovan BM, Semmens R, Keck P, Brimhall E, Busch K, Weindling M, et al. Toward a more humane genetics education: Learning about the social and quantitative complexities of human genetic variation research could reduce racial bias in adolescent and adult populations. Science Education. 2019;103(3):529–60.

[7] Hornsey MJ, Lewandowsky S. A toolkit for understanding and addressing climate scepticism. Nature Human Behaviour. 2022;6(11):1454–64. doi: 10.1038/s41562-022-01463-y 36385174 PMC7615336

[8] Dubay WH. Smart Language: Readers, Readability, and the Grading of Text 2007.

[9] Bullock OM, Colón Amill D, Shulman HC, Dixon GN. Jargon as a barrier to effective science communication: Evidence from metacognition. Public Understanding of Science. 2019;28(7):845–53. doi: 10.1177/0963662519865687 31354058

[10] Sharon AJ, Baram-Tsabari A. Measuring mumbo jumbo: A preliminary quantification of the use of jargon in science communication. Public Understanding of Science. 2014;23(5):528–46. doi: 10.1177/0963662512469916 23825277

[11] Akin H, Landrum A. A recap: Heuristics, biases, values, and other challenges to communicating science. The Oxford Handbook of the Science of Science Communication 2017. p. 455–60.

[12] Condit CM. Laypeople Are Strategic Essentialists, Not Genetic Essentialists. 2019. Report No.: 0093–0334 Contract No.: 3. 10.1002/hast.1014 31268567

[13] Carver R, Waldahl R, Breivik J. Frame that gene. EMBO Reports. 2008;9(10):943–7. 18772895 10.1038/embor.2008.176 PMC2572113

[14] Hase V, Schäfer MS. Big data and computational methods: Methodological advances for analyzing mediated environmental communication. The Routledge Handbook of Environment and Communication: Routledge; 2022. p. 239–52.

[15] Edwards ML, Ziegler C. Examining science communication on Reddit: From an “Assembled” to a “Disassembling” approach. Public Understanding of Science. 2022;31(4):473–88. doi: 10.1177/09636625211057231 35023409

[16] Guenther L, Wilhelm C, Oschatz C, Brück J. Science communication on Twitter: Measuring indicators of engagement and their links to user interaction in communication scholars’ Tweet content. Public Understanding of Science. 2023:09636625231166552. doi: 10.1177/09636625231166552 37132036 PMC10552346

[17] Welbourne DJ, Grant WJ. Science communication on YouTube: Factors that affect channel and video popularity. Public Understanding of Science. 2016;25(6):706–18. doi: 10.1177/0963662515572068 25698225

[18] Winters M, Larsson A, Kowalski J, Sundberg CJ. The association between quality measures of medical university press releases and their corresponding news stories—Important information missing. PLoS One. 2019;14(6):e0217295. doi: 10.1371/journal.pone.0217295 31188838 PMC6561540

[19] Schäfer MS, Hase V. Computational methods for the analysis of climate change communication: Towards an integrative and reflexive approach. Wiley Interdisciplinary Reviews: Climate Change. 2022;14(2):e806.

[20] Boumans JW, Trilling D. Taking stock of the toolkit: An overview of relevant automated content analysis approaches and techniques for digital journalism scholars. Digital Journalism. 2016;4(1):8–23.

[21] Christensen B, Laydon D, Chelkowski T, Jemielniak D, Vollmer M, Bhatt S, et al. Quantifying changes in vaccine coverage in mainstream media as a result of the COVID-19 outbreak: Text mining study. JMIR infodemiology. 2022;2(2):e35121. doi: 10.2196/35121 36348981 PMC9631944

[22] Zolnoori M, Huang M, Patten CA, Balls-Berry JE, Goudarzvand S, Brockman TA, et al. Mining news media for understanding public health concerns. Journal of Clinical and Translational Science. 2021;5(1):e1. 10.1017/cts.2019.434 PMC8057471 33948233

[23] Sun Y. A text mining approach to analyze public media science coverage and public interest in science. International Journal of Machine Learning and Computing. 2014;4(6):496.

[24] Petersen A. Biofantasies: genetics and medicine in the print news media. Social Science & Medicine. 2001;52(8):1255–68. doi: 10.1016/s0277-9536(00)00229-x 11281408

[25] Hjörleifsson S, Árnason V, Schei E. Decoding the genetics debate: Hype and hope in Icelandic news media in 2000 and 2004. New Genetics and Society. 2008;27(4):377–94.

[26] Conrad P. Genetics and Behavior in the news: Dilemmas if a raising paradigm. In: Alper CA J. S., Asch A., Beckwith J., Conrad P. & Geller L. N., editor. The Double-Edged Helix: Implications of Genetics in a Diverse Society. Baltimore, MD: John Hopkins University Press; 2002.

[27] Wilde A, Bonfiglioli C, Meiser B, Mitchell PB, Schofield PR. Portrayal of psychiatric genetics in Australian print news media, 1996–2009. The Medical Journal of Australia. 2011;195(7):401–4. doi: 10.5694/mja10.10167 21978348

[28] Genetics ASoH. Public Attitudes Toward Genetics & Genomics Research. 2020.

[29] Condit CM, Ofulue N, Sheedy KM. Determinism and mass-media portrayals of genetics. The American Journal of Human Genetics. 1998;62(4):979–84. doi: 10.1086/301784 9529342 PMC1377024

[30] Carver RB, Rødland EA, Breivik J. Quantitative frame analysis of how the gene concept is presented in tabloid and elite newspapers. Science Communication. 2013;35(4):449–75.

[31] Abdellaoui A, Yengo L, Verweij KJ, Visscher PM. 15 years of GWAS discovery: Realizing the promise. The American Journal of Human Genetics. 2023. doi: 10.1016/j.ajhg.2022.12.011 36634672 PMC9943775

[32] Buniello A, MacArthur JAL, Cerezo M, Harris LW, Hayhurst J, Malangone C, et al. The NHGRI-EBI GWAS Catalog of published genome-wide association studies, targeted arrays and summary statistics 2019. Nucleic Acids Research. 2019;47(D1):D1005–D12. doi: 10.1093/nar/gky1120 30445434 PMC6323933

[33] World Health Organization. International Statistical Classification of Diseases and Related Health Problems (10th Revision). 2016 [Available from: https://icd.who.int/browse10/2016/en.

[34] Altmetric. Getting Started with the Altmetric Explorer API 2021 [Available from: https://api.altmetric.com/.

[35] Baessa M, Lery T, Grenz D, Vijayakumar J. Connecting the pieces: Using ORCIDs to improve research impact and repositories. F1000Research. 2015;4. 10.12688/f1000research.6502.1 PMC4654437 26664706

[36] Ortega JL. Reliability and accuracy of altmetric providers: a comparison among Altmetric. com, PlumX and Crossref Event Data. Scientometrics. 2018;116(3):2123–38.

[37] Silge J, Robinson D. tidytext: Text mining and analysis using tidy data principles in R. Journal of Open Source Software. 2016;1(3):37.

[38] R Core Team. R: A language and environment for statistical computing. Vienna, Austra: R Foundation for Statistical Computing; 2020.

[39] Chang W, Cheng J, Allaire J, Xie Y, McPherson J. Shiny: web application framework for R. R package version 1.4.0. 2019.

[40] Morosoli JJ. NeWAS browser 2021 [Available from: https://jjmorosoli.shinyapps.io/newas/.

[41] National Human Genome Research Institute. Talking Glossary of Genetic Terms 2020 [Available from: https://www.genome.gov/genetics-glossary.

[42] Carver RB, Wiese EF, Breivik J. Frame analysis in science education: A classroom activity for promoting media literacy and learning about genetic causation. International Journal of Science Education, Part B. 2014;4(3):211–39.

[43] Liu B. Sentiment Analysis and Opinion Mining. Cham: Springer International Publishing; 2012.

[44] Roberts ME, Stewart BM, Tingley D. stm: R package for structural topic models. Journal of Statistical Software. 2014;10(2):1–40.

[45] Mimno D, Wallach HM, Talley E, Leenders M, McCallum A, editors. Optimizing semantic coherence in topic models. Proceedings of the conference on empirical methods in natural language processing; 2011: Association for Computational Linguistics.

[46] Journal Citation Reports [Internet]. 2019 [cited 10/2/2018]. Available from: https://jcr.clarivate.com/.

[47] Brooks ME, Kristensen K, van Benthem KJ, Magnusson A, Berg CW, Nielsen A, et al. glmmTMB balances speed and flexibility among packages for zero-inflated generalized linear mixed modeling. The R Journal. 2017;9(2):378–400.

[48] Scribblrs. The Science Behind Buzzfeed’s Viral Articles Scribblrs 2016 [Available from: http://www.scribblrs.com/science-behind-buzzfeeds-viral-articles/.

[49] Cheng C, Dunn M. Health literacy and the Internet: a study on the readability of Australian online health information. Australian and New Zealand Journal of Public Health. 2015;39(4):309–14. doi: 10.1111/1753-6405.12341 25716142

[50] National Cancer Institute. Making Health Communication Programs Work: A Planner’s Guide 2004. Available from: https://www.cancer.gov/publications/health-communication/pink-book.pdf.

[51] U.S. Census Bureau. American Community Survey: 2018 2019 [Available from: https://data.census.gov/cedsci/.

[52] Heine SJ, Dar-Nimrod I, Cheung BY, Proulx T. Essentially biased: Why people are fatalistic about genes. Advances in Experimental Social Psychology. 55: Elsevier; 2017. p. 137–92.

[53] Cimpian A, Salomon E. The inherence heuristic: An intuitive means of making sense of the world, and a potential precursor to psychological essentialism. Behavioral and Brain Sciences. 2014;37(5):461–80. doi: 10.1017/S0140525X13002197 24826999

[54] Zimmermann B, Elger B, Shaw D. Media coverage of ethical issues in predictive genetic testing: a qualitative analysis. AJOB Empirical Bioethics. 2019;10(4):250–64. doi: 10.1080/23294515.2019.1670275 31596686

[55] Lewis CM, Vassos E. Polygenic scores in psychiatry: on the road from discovery to implementation. American Journal of Psychiatry. 2022;179(11):800–6. doi: 10.1176/appi.ajp.20220795 36317334

[56] Callaham M, Wears RL, Weber E. Journal prestige, publication bias, and other characteristics associated with citation of published studies in peer-reviewed journals. JAMA. 2002;287(21):2847–50. doi: 10.1001/jama.287.21.2847 12038930

[57] Dumas-Mallet E, Garenne A, Boraud T, Gonon F. Does newspapers coverage influence the citations count of scientific publications? An analysis of biomedical studies. Scientometrics. 2020;123:413–27.

[58] McKiernan EC, Schimanski LA, Muñoz Nieves C, Matthias L, Niles MT, Alperin JP. Use of the Journal Impact Factor in academic review, promotion, and tenure evaluations. eLife. 2019;8:e47338. doi: 10.7554/eLife.47338 31364991 PMC6668985

